# Thigh length versus knee length antiembolism stockings for the prevention of deep vein thrombosis in postoperative surgical patients; a systematic review and network meta-analysis

**DOI:** 10.1136/bmjopen-2015-009456

**Published:** 2016-02-16

**Authors:** Ros Wade, Fiona Paton, Stephen Rice, Gerard Stansby, Peter Millner, Hayley Flavell, Dave Fox, Nerys Woolacott

**Affiliations:** 1 Centre for Reviews and Dissemination, University of York, York, UK; 2 Northern Vascular Centre, Newcastle upon Tyne Hospitals NHS Foundation Trust, Newcastle upon Tyne, UK; 3 Department of Spinal Surgery, Leeds Teaching Hospitals NHS Trust, Leeds, UK; 4 Department of Pathology, Royal Bournemouth and Christchurch Hospitals NHS Foundation Trust, Bournemouth, UK

**Keywords:** SURGERY, VASCULAR MEDICINE

## Abstract

**Objectives:**

To assess the clinical effectiveness of thigh length versus knee length antiembolism stockings for the prevention of deep vein thrombosis (DVT) in surgical patients.

**Design:**

Systematic review and meta-analysis using direct methods and network meta-analysis.

**Methods:**

Previous systematic reviews and electronic databases were searched to February 2014 for randomised controlled trials (RCTs) of thigh length or knee length antiembolism stockings in surgical patients. Study quality was assessed using the Cochrane Risk of Bias Tool. The primary outcome was incidence of DVT. Analysis of the DVT data was performed using ORs along with 95% CIs. The I^2^ statistic was used to quantify statistical heterogeneity.

**Results:**

23 RCTs were included; there was substantial variation between the trials and many were poorly reported with an unclear risk of bias. Five RCTs directly comparing thigh length versus knee length stockings were pooled and the summary estimate of effect favouring thigh length stockings was not statistically significant (OR 1.48, 95% CI 0.80 to 2.73). 13 RCTs were included in the network meta-analysis; thigh length stockings with pharmacological prophylaxis were more effective than knee length stockings with pharmacological prophylaxis, but again results were not statistically significant (OR 1.76, 95% credible intervals 0.82 to 3.53).

**Conclusions:**

Thigh length stockings may be more effective than knee length stockings, but results did not reach statistical significance and the evidence base is weak. Further research to confirm this finding is unlikely to be worthwhile. While thigh length stockings appear to have superior efficacy, practical issues such as patient acceptability may prevent their wide use in clinical practice.

**Systematic review registration number:**

CRD42014007202.

**Strengths and limitations of this study**

- This systematic review used all the available randomised evidence on thigh length or knee length antiembolism stockings to indirectly compare the two stocking lengths.
- Many trials were old and poorly reported and there was substantial variation in terms of patient characteristics and interventions used.
- Standard meta-analysis and network meta-analysis were undertaken in order to compare all relevant treatments with one another.
- The results of the network meta-analysis were consistent with the direct meta-analysis, although there was significant statistical heterogeneity in the models.
- The uncertain quality of many of the included trials reduces the reliability of the results of the review.

## Introduction

Deep vein thrombosis (DVT) is a condition in which a blood clot forms in one of the deep veins of the body, usually in the leg. An emboli is formed if the blood clot or part of the blood clot detaches and travels through the venous system. If the clot lodges in the lung, this is termed a pulmonary embolism (PE) and this may be fatal. DVT and PE are collectively known as venous thromboembolism (VTE). The House of Commons Health Committee reported in 2005 that an estimated 25 000 people in the UK die each year from potentially preventable hospital-acquired VTE.^1^

Surgical patients are at an increased risk of developing DVT, due to stasis in venous blood flow and increased coagulability of the blood, caused by factors such as immobilisation, decreased fluid intake and blood or body fluid loss. It has been estimated that between 45 and 51% of patients undergoing orthopaedic surgery develop DVT, if not provided with adequate prophylaxis.^1^ Routine prophylaxis reduces morbidity, mortality and health service costs in patients at risk.^2^ Prophylaxis can be pharmacological (such as low-molecular-weight heparin (LMWH)) and/or mechanical (such as antiembolism stockings (also known as graduated compression stockings)).

Antiembolism stockings are available as thigh length or knee length stockings. They exert graded pressure at a decreasing gradient from the ankle towards the thigh or knee, which increases blood flow velocity and promotes venous return. Patients have reported that both thigh length and knee length stockings are difficult to use, but fewer patients reported discomfort with knee length stockings and patients are more likely to wear knee length stockings correctly.^3–5^

The National Institute for Health and Care Excellence (NICE) guideline ‘Venous thromboembolism: reducing the risk’ (CG92) states that the length of stockings is a controversial issue and there is no clear randomised evidence that one length is more effective than another.^6^ The Scottish Intercollegiate Guidelines Network (SIGN) guideline on the prevention and management of VTE (SIGN guideline 122) states that studies comparing above-knee with below-knee stockings have been too small to determine whether or not they are equally effective.^2^

This systematic review aims to address this question more definitively by utilising all the available randomised evidence on thigh length or knee length stockings, rather than just trials that directly compare the two stocking lengths: using both standard meta-analysis and network meta-analysis. Network meta-analysis enables a comparison of all relevant treatments with one another. This review was undertaken as part of a larger research project to establish the expected value (cost-effectiveness) of undertaking additional research comparing the relative effectiveness of the two different lengths of stocking, in addition to standard pharmacoprophylaxis.^7^

## Methods

We conducted a systematic review to assess the clinical effectiveness of thigh length versus knee length antiembolism stockings for the prevention of DVT in surgical patients. Owing to the anticipated paucity of research evidence directly comparing thigh length stockings with knee length stockings, we also sought studies comparing thigh length stockings with a control treatment and studies comparing knee length stockings with a control treatment.

Clinical advice was provided by an advisory group which included a vascular surgeon, an orthopaedic surgeon and an anticoagulant and thrombosis consultant nurse. A patient representative also provided information on her experiences of using antiembolism stockings after two different types of surgery.

The research protocol was registered on the international prospective register of systematic reviews (PROSPERO registration number: CRD42014007202).

### Search strategy

Eleven guideline and systematic review databases (including the Cochrane Database of Systematic Reviews, Database of Abstracts of Reviews of Effects, PROSPERO, Health Technology Assessment Database and National Guidelines Clearinghouse) were searched up to August 2013 for reviews of antiembolism stockings. The included and excluded studies listed by relevant systematic reviews were screened for relevant primary studies. To update the searches undertaken in the relevant reviews, systematic searches for RCTs published since January 2010 were undertaken in February 2014. Six electronic sources were searched (MEDLINE, MEDLINE In-Process, EMBASE, CINAHL, AMED and CENTRAL) as well as two grey literature databases (ClinicalTrials.gov and Current Controlled Trials). No language restrictions were applied. In addition, clinical advisors were consulted for additional potentially relevant studies and reference lists of all included studies were manually searched. Records were inserted into an EndNote library.

The search strategy developed for Ovid MEDLINE is presented below.

Ovid MEDLINE(R) In-Process & Other Non-Indexed Citations and Ovid MEDLINE(R), 1946 to Present. Searched on 19 February 2014. Date limited to 2010 onwards. Search strategy:

1. exp “embolism and thrombosis”/ (172610)
2. (thrombos$ or thrombus$ or thrombotic or thrombolic$ or thromboemboli$ or thromboprophyla$ or embol$).ti,ab. (232741)
3. (DVT$ or PE or PTS).ti,ab. (34899)
4. 1 or 2 or 3 (317779)
5. Stockings, Compression/ or Compression Bandages/ (1165)
6. (stocking$ or hose or hosiery or tights or sock$ or TEDS).ti,ab. (10451)
7. (compression adj3 bandage$).ti,ab. (486)
8. 5 or 6 or 7 (11541)
9. 4 and 8 (1418)
10. randomized controlled trial.pt. (362662)
11. controlled clinical trial.pt. (87530)
12. randomized.ab. (282970)
13. placebo.ab. (149727)
14. drug therapy.fs. (1661607)
15. randomly.ab. (205717)
16. trial.ab. (291784)
17. groups.ab. (1315795)
18. 10 or 11 or 12 or 13 or 14 or 15 or 16 or 17 (3250729)
19. 9 and 18 (518)
20. limit 19 to yr=“2010 -Current” (141).

### Study selection

RCTs assessing thigh length or knee length antiembolism stockings (with or without standard pharmacological prophylaxis) in surgical patients were eligible for inclusion; the length of stocking had to be clearly stated. The primary outcome was incidence of DVT; DVT data were included only if definitively diagnosed using radioiodine (125I) fibrinogen uptake, venography, Doppler ultrasound or MRI. Studies reporting complications and consequences associated with DVT (such as the incidence of PE, incidence of post-thrombotic syndrome and mortality) or adverse effects related to the use of antiembolism stockings were also included.

Studies identified by the searches were independently assessed for inclusion by two reviewers using the prespecified inclusion criteria stated above. Disagreements were resolved through discussion and, where necessary, by consultation with a third reviewer.

### Data extraction

Data extraction was conducted by one reviewer using a piloted and standardised data extraction form in Eppi-Reviewer 4.0 and independently checked by a second reviewer. Discrepancies were resolved by discussion, with involvement of a third reviewer when necessary. In cases where the same study was reported in multiple publications, the most up to date or comprehensive publication was used for data extraction. Data were extracted on study details (eg, author, year, location of study), patient characteristics (eg, age, gender, type of surgery, baseline risk factors for VTE), details of the intervention (eg, type of stocking, duration of use, co-interventions including pharmacological thromboprophylaxis), and reported outcomes (eg, method of assessment and results).

### Quality assessment

The quality of the individual trials was assessed by one reviewer, and independently checked by a second reviewer; disagreements were resolved by consensus and if necessary a third reviewer was consulted. The quality of included trials was assessed using the Cochrane Risk of Bias Tool, which assesses methods of randomisation and allocation concealment, blinding, completeness of outcome data and selective outcome reporting.^8^ Similarity of treatment groups at baseline was also assessed. Each trial was given an overall risk of bias judgement; trials that had a low risk of bias for all key domains were judged to have a low overall risk of bias, trials that had a high risk of bias for one or more key domains were judged to have a high overall risk of bias, and trials that had an unclear risk of bias for one or more key domains were judged to have an unclear overall risk of bias.

### Synthesis

Analysis of the DVT data was performed using ORs along with 95%CIs. Owing to the clinical and methodological variation between trials a random effects model was used to pool data. The I^2^ statistic was used to quantify statistical heterogeneity. The statistical package used for analysis was RevMan V.5.2.

A network meta-analysis (NMA) was performed to investigate whether the utilisation of indirect evidence would increase the precision of the relative effect estimate for thigh length versus knee length stockings. It also provides an estimate of the relative effect of all treatments relative to one another. A high level of inconsistency between the direct and indirect evidence suggests clinical or methodological heterogeneity, which increases the uncertainty in the effect estimates. Although several outcomes were investigated in the review, there was only sufficient evidence to perform an NMA for the outcome DVT. To create the network, interventions that were considered sufficiently similar relative to the interventions of interest were lumped together: the effectiveness of LMWH, low dose heparin and fondaparinux were assumed to be the same, and these were therefore lumped together in the network and were referred to collectively as ‘heparin’. Based on the advice of the clinical advisors, it was assumed that there was no stocking-heparin interaction in the base case analysis, that is, the effect of thigh length stockings compared to knee length stockings is the same as thigh length stockings plus concomitant heparin compared to knee length stockings plus concomitant heparin. This assumption was tested in a sensitivity analysis. A random effects analysis was used and credible intervals (CrI) represent the uncertainty around the average treatment effect across trials. The only potential effect modifier for which there was evidence across the trials and a relevant network, was whether or not patients had undergone orthopaedic surgery, which carries a high risk of DVT. Therefore, a subgroup analysis was conducted to compare the effectiveness of antiembolism stockings in orthopaedic surgery patients versus other surgery patients. The model, written in WinBUGS, was based on code presented in the NICE Technical Support Document 2.^9^

Data on the incidence of PE, mortality and adverse events related to the use of antiembolism stockings were tabulated and synthesised narratively.

## Results

During protocol development, scoping searches identified two particularly relevant Cochrane reviews.^10^ ^11^ Therefore, many relevant trials were identified from the included and excluded studies lists of these reviews (among others), prior to running the update searches for primary studies.

The electronic search of the relevant systematic review and guideline databases identified 307 records, of which 12 appeared to be systematic reviews of antiembolism stockings in postoperative surgical patients (including the two reviews identified during the protocol development stage). These reviews were obtained so that their lists of included and excluded studies could be systematically searched for potentially relevant primary studies. A total of 137 records were added to the EndNote library from the included and excluded studies lists of the 12 relevant systematic reviews (after removal of duplicates). The update searches of electronic databases (from 2010 to February 2014) identified an additional 330 records, which were also added to the EndNote library.

**Figure 1:**
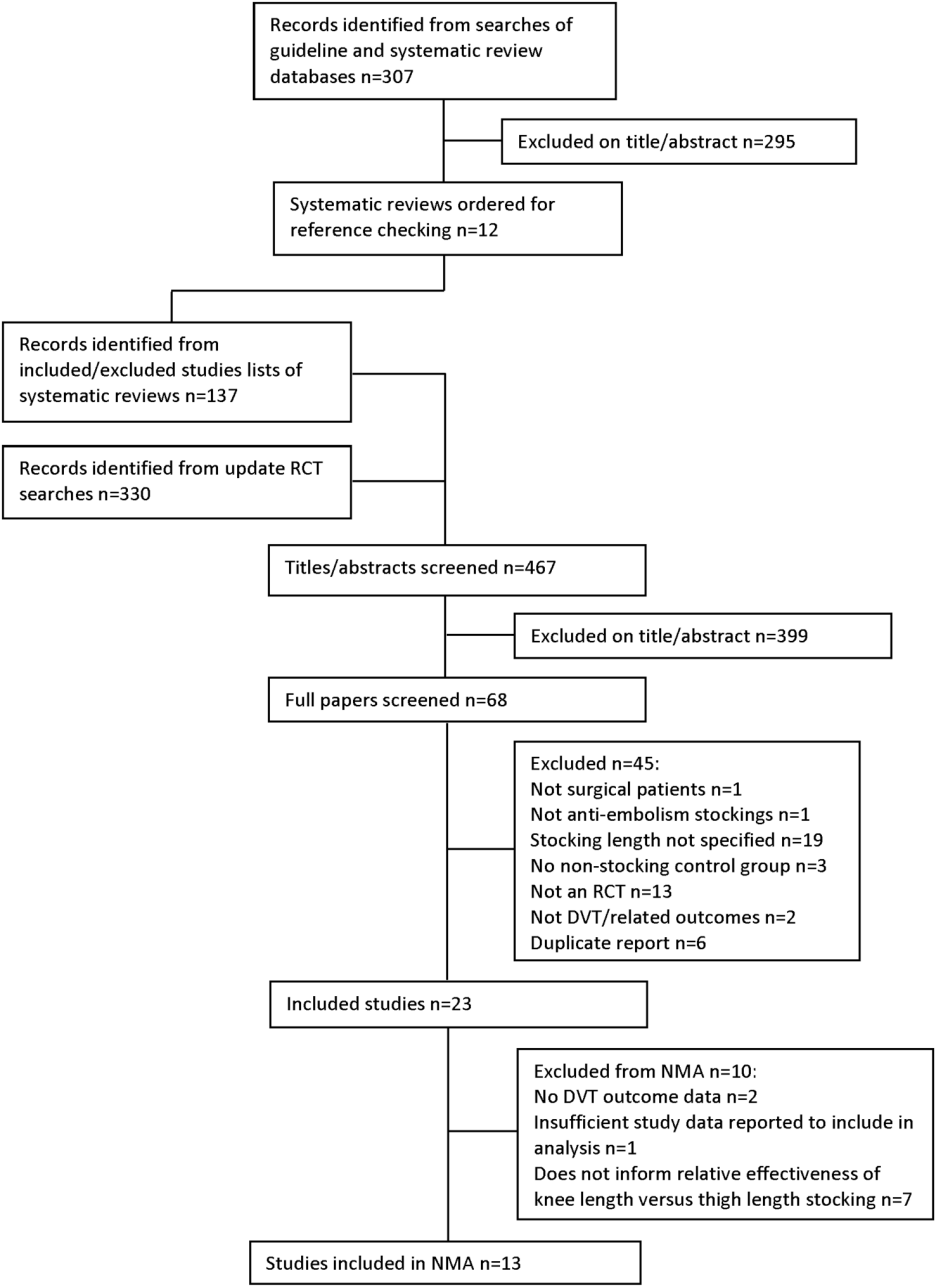
Flow diagram of the study selection process. DVT, deep vein thrombosis; NMA, network meta-analysis; RCT, randomised controlled trial.

The full papers of 68 potentially relevant primary studies were screened for inclusion in the review. Twenty-three RCTs met the inclusion criteria and were included in the systematic review (see online supplementary tables S1–5 for study details).^12–34^ Figure 1 presents the flow of studies through the study selection process.

**Figure 2:**
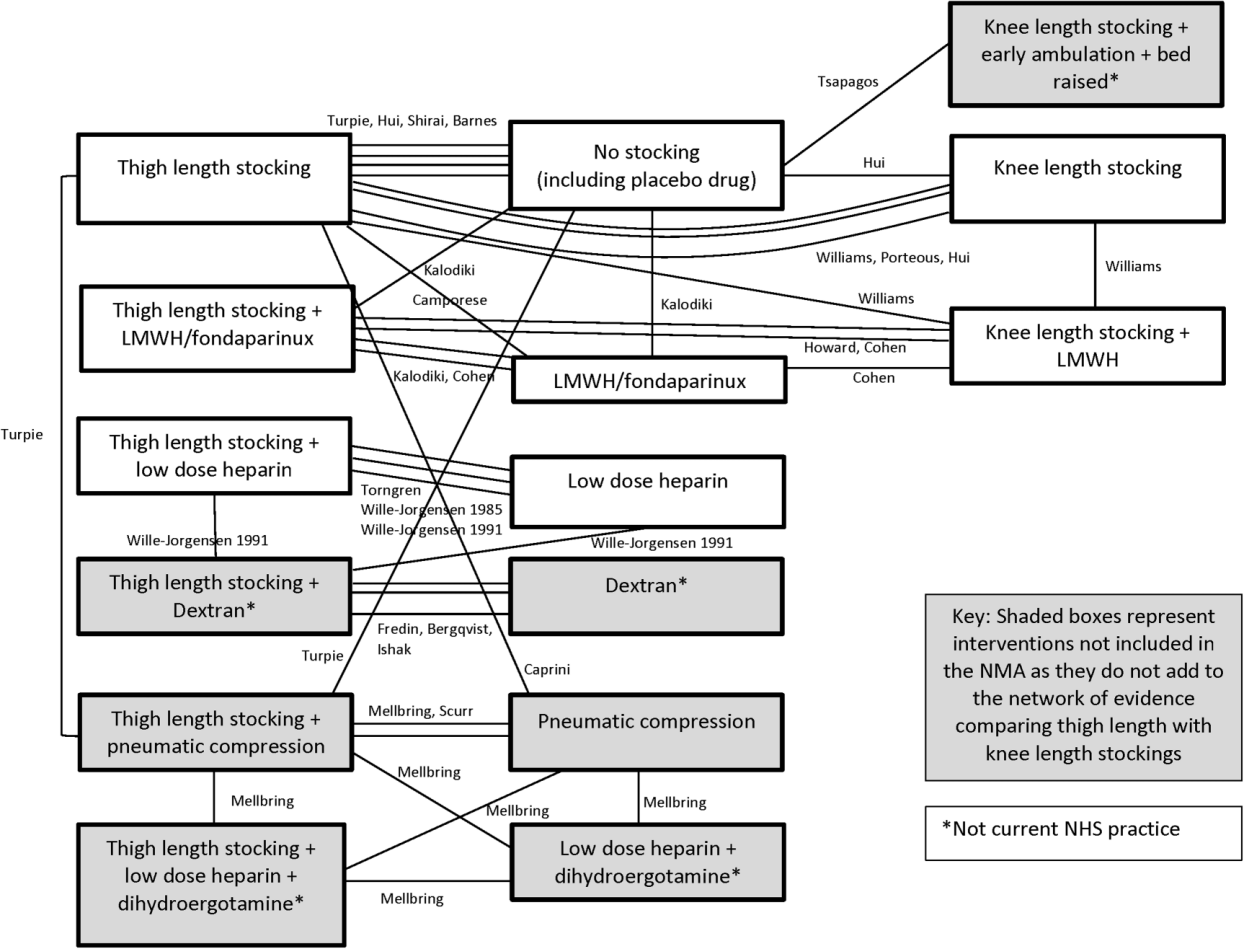
Network of trials presenting data on DVT. DVT, deep vein thrombosis; LMWH, low-molecular-weight heparin; NHS, National Health Service; NMA, network meta-analysis.

Of these 23 RCTs, 21 reported data for the outcome DVT. However, one trial did not report sufficient data to be included in the meta-analysis or NMA, as total numbers of patients in treatment groups were not reported.^34^ Figure 2 shows the network of 20 trials that presented adequate data on DVT.

Seven trials did not add to the network of evidence comparing thigh length with knee length antiembolism stockings: these trials compared thigh length or knee length stockings with a different intervention, such as pneumatic compression or dextran.^14^ ^16^ ^19^ ^24^ ^27^ ^28^ ^31^ Both thigh length stockings and knee length stockings needed to be compared with a common comparator to be able to inform the relative effectiveness of the two different stocking lengths. Therefore, 13 RCTs contained data that directly or indirectly informed the relative effectiveness of thigh length versus knee length stockings and were included in the standard meta-analysis or NMA or both.^12^ ^13^ ^15^ ^17^ ^18^ ^20–23^ ^25^ ^29^ ^32^ ^33^

There was substantial variation between the 23 included trials in terms of the patient characteristics, suggesting that the participants had a different baseline risk for DVT. There was also variation in the interventions used in the RCTs; in some trials a stocking was only worn on one leg, rather than both legs, and the duration of use varied between trials. Concomitant pharmacological prophylaxis also varied between trials.

Generally the trial methods were poorly reported, with a high proportion of assessments for each quality domain having to be recorded as unclear. Overall 3 RCTs can be considered to have a low risk of bias,^14^ ^23^ ^25^ 5 have a high risk of bias^18^ ^22^ ^27^ ^32^ ^33^ and for 15 RCTs the reporting was inadequate to judge the risk of bias.^12^ ^13^ ^15–17^ ^19–21^ ^24^ ^26^ ^28–31^ ^34^

Many of the included RCTs dated back to the 1970s^13^ ^19^ and 1980s,^14^ ^16–18^ ^20^ ^22^ ^24^ ^26–31^ ^33^ therefore, their results may not be generalisable to current practice; surgical practice has changed over time with less invasive surgical procedures, shorter duration of hospitalisation and earlier mobilisation after surgery.

### DVT results

Twenty RCTs reported rates of DVT and provided sufficient data to be included in meta-analyses. Where reported, the majority of DVTs were asymptomatic, the clinical consequences of which are unknown.

#### Thigh length stockings (with or without pharmacological prophylaxis) versus knee length stockings (with or without pharmacological prophylaxis)

Two RCTs^12^ ^25^ directly compared thigh length versus knee length stockings, plus pharmacological prophylaxis, reflecting current practice for the treatment of patients at high risk of DVT; the results were inconsistent in terms of the direction of effect. The reasons for the inconsistent findings between the two trials were unclear and may be due to chance.

Four additional RCTs that compared thigh length versus knee length stockings were identified, but these trials did not include additional pharmacological prophylaxis.^29^ ^32–34^ Unfortunately, the trial by Ayhan (2013) was reported only as an abstract and did not provide details on the number of patients in each treatment group; therefore this trial was excluded from meta-analyses.^34^

**Figure 3:**
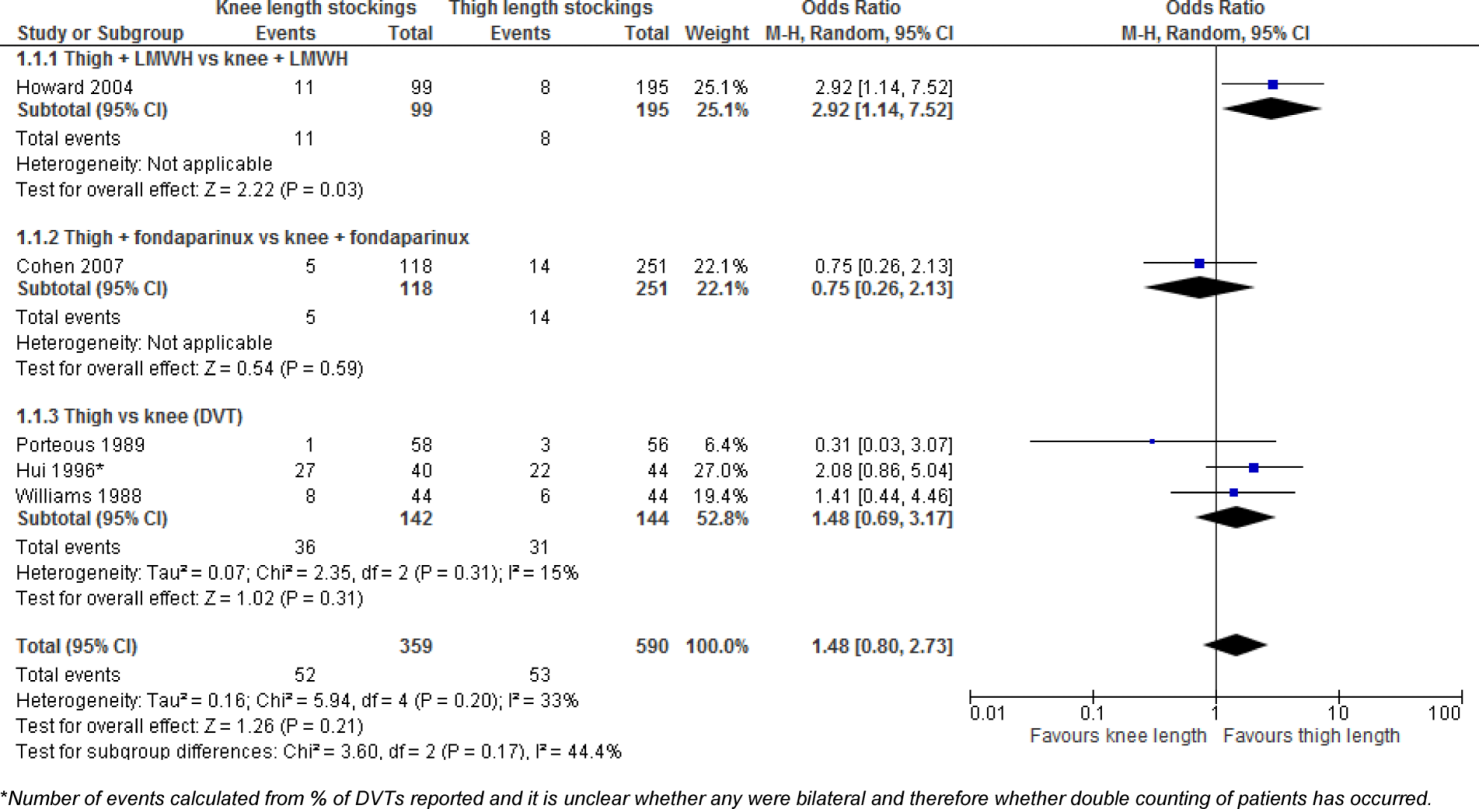
Rates of DVT (or VTE) comparing thigh length stockings (with or without pharmacological prophylaxis) versus knee length stockings (with or without pharmacological prophylaxis). DVT, deep vein thrombosis; VTE, venous thromboembolism.

The five available RCTs comparing thigh length versus knee length stockings with or without additional pharmacological prophylaxis were combined using meta-analysis (figure 3); the summary estimate of effect indicated a trend favouring thigh length stockings, but the findings were not statistically significant (knee vs thigh OR 1.48, 95% CI 0.80 to 2.73, p=0.21; I^2^=33%).

There was some inconsistency in the direction of effect for trials assessing patients in similar surgical groups. Cohen *et al*^25^ and Hui *et al* 32 included orthopaedic patients, and Porteous *et al*^29^ and Williams *et al*^33^ included patients undergoing abdominal surgery. The reasons for the inconsistency were unclear and may be due to chance.

The other 15 RCTs that reported DVT results compared either thigh length or knee length antiembolism stockings with no stocking or with another method of thromboprophylaxis, therefore their results do not directly inform the comparison of thigh versus knee length stockings.

### Network meta-analysis

Thirteen trials contained data that directly or indirectly informed the relative effectiveness of thigh length versus knee length stockings with or without pharmacological prophylaxis for the prevention of DVT and were included in the NMA. Table 1 presents the direct comparisons included in the NMA, and the number of studies reporting that direct comparison. The number of direct comparisons, 19, is greater than the number of studies in the NMA because three three-armed trials were included in the analysis.

**Table 1:** The direct comparisons included in the network meta-analysis, and the number of studies reporting that direct comparison

Treatment	Knee length stocking	No prophylaxis	Knee length stocking plus heparin	Heparin
Thigh length stocking	3	4	1	1
Knee length stocking		1	1	–
Thigh length stocking plus heparin			2	5
Knee length stocking plus heparin				1

The results of the NMA are the estimates of the average effects across a heterogeneous set of trials. The credible intervals (CrI) presented represent the uncertainty around that average. There was significant statistical heterogeneity in the models and inconsistency indicating that there may be underlying unknown clinical and methodological heterogeneity across the trials.

The results of the base case analysis found that heparin was statistically significantly more effective than no treatment (median OR 0.26, 95% CrI 0.09 to 0.87, p=0.03), thigh length stockings with heparin were statistically significantly more effective than heparin alone (median OR 0.38, 95% CrI 0.21 to 0.63, p=0.00) and knee length stockings with heparin were more effective than heparin alone, although this result was not statistically significant (median OR 0.68, 95% CrI 0.27 to 1.38, p=0.28).

In the base case analysis, thigh length stockings with pharmacological prophylaxis were more effective than knee length stockings with pharmacological prophylaxis (knee vs thigh OR 1.76, 95% CrI 0.82 to 3.53, p=0.12), but this result was not statistically significant. The indirect estimate favours thigh length stockings slightly more than the direct estimate of 1.48 (95% CI 0.80 to 2.73, p=0.21) from the direct meta-analysis presented above, but there is also greater uncertainty in the estimate. The NMA did not increase the precision of the relative effect estimate for thigh length versus knee length stockings because of the uncertainty associated with the inconsistency between direct and indirect estimates of effect. The full table of results in the base case are presented in online supplementary table S6.

The effectiveness of each treatment is represented by the absolute risk of DVT in table 2. The baseline risk of DVT (both symptomatic and asymptomatic) for moderate risk general surgical patients taking heparin was estimated to be 9.88%, estimated using the American College of Chest Physicians Guidelines for the prevention of VTE in non-orthopaedic surgical patients.^35^ Using this baseline risk estimate, the absolute risks of DVT for patients using the different treatments are presented below in table 2. The combination of thigh length stockings with pharmacological prophylaxis was the most effective treatment with an absolute risk of DVT of 4.04%. The probability that thigh length stockings with pharmacological prophylaxis is the most effective treatment in a new trial of all the treatments is 73%, as displayed in table 2. The probability of being the most effective treatment does not simply reflect the effectiveness of the treatment, but also the uncertainty in the estimate. While thigh length stockings plus pharmacological prophylaxis appear to be the most effective treatment with little uncertainty, the marginal benefit of thigh length stockings plus heparin over heparin alone is less than the marginal benefit of heparin over no treatment as heparin has already reduced the risk of DVT substantially.

**Table 2:** Probability of being the most effective treatment in a new trial of all treatments

Treatment	Probability of being the most effective treatment	Absolute risk of DVT (%)
No treatment	0.00	29.28
Thigh length stocking	0.04	13.76
Knee length stocking	0.02	22.01
Heparin	0.02	9.88
Thigh length stocking plus heparin	0.73	4.04
Knee length stocking plus heparin	0.20	6.94

DVT, deep vein thrombosis.

The sensitivity analysis modelling an interaction between thigh or knee length stockings and heparin produced results with the same direction of effect but greater uncertainty in the effect estimate (knee vs thigh OR 2.59, CrI 0.92 to 7.84, p=0.10). The subgroup analysis suggested that thigh length stockings with heparin appear to be more effective in the non-orthopaedic surgery group than in the orthopaedic surgery group. The median ORs are slightly more in favour of both thigh and knee length stockings with heparin compared to heparin alone for the non-orthopaedic surgery group (thigh: median OR 3.83, 95% CrI 2.29 to 6.66, p=0.00; knee: median OR 2.16, 95% CrI 0.90 to 5.21, p=0.09) compared to the orthopaedic surgery group (thigh: median OR 2.05, 95% CrI 1.32 to 3.23, p=0.00; knee: median OR 1.32, 95% CrI 0.72 to 2.46, p=0.37).

### PE, mortality and adverse event results

Fifteen RCTs assessed PE or fatal PE, 11 RCTs assessed mortality and 12 RCTs reported results relating to adverse events. PE events and VTE-related deaths were generally rare in the included trials. Adverse events were rarely reported and those related to antiembolism stockings were minor events, including minor foot abrasions, superficial thrombophlebitis or the stocking slipping down. The majority of complications reported were minor bleeding complications associated with pharmacoprophylaxis, although the proportion of patients reporting such events was low; between 1% and 4%.

## Discussion

This systematic review assessed the clinical effectiveness of thigh length versus knee length antiembolism stockings for the prevention of DVT in surgical patients. The review only included studies of surgical patients; therefore, the results are not generalisable to other patient populations, who may have a different baseline risk of DVT and of the adverse effects of thromboprophylaxis. Patients with stroke have been investigated separately in a large RCT of thigh length versus knee length antiembolism stockings, which found that DVT occurred more often in patients who wore knee length stockings than those who wore thigh length stockings.^36^

A previous Cochrane review comparing knee length versus thigh length antiembolism stockings in postoperative surgical patients included three of the five RCTs included in our direct meta-analysis.^11^ The Cochrane review also found no statistically significant difference in clinical effectiveness between the two stocking lengths in terms of reducing the incidence of DVT. The authors concluded that there was insufficient high quality evidence to determine whether thigh length or knee length stockings differ in their effectiveness in terms of reducing the incidence of DVT in hospital in patients.

Our systematic review included a network meta-analysis of all the trials that indirectly informed the relative effectiveness of thigh length versus knee length antiembolism stockings, with or without pharmacological prophylaxis, for the prevention of DVT in surgical patients. The results of the NMA were consistent with the direct meta-analysis, without increasing the precision of the estimates. Overall, thigh length stockings with pharmacological prophylaxis appears to be the most effective method of preventing DVT in surgical patients, the NMA results also indicate that the marginal benefit of thigh length stockings plus heparin over heparin alone is less than the marginal benefit of heparin over no treatment, as heparin already reduces the risk of DVT substantially.

Evidence relating to other outcomes was sparse; few trials reported complications and consequences associated with DVT, such as the incidence of PE, post-thrombotic syndrome and mortality or adverse effects.

Despite the weak evidence base and importance of the question, it is unlikely to be worthwhile undertaking a new definitive trial comparing thigh length versus knee length antiembolism stockings. Such a trial would need to be very large to enable assessment of clinically relevant DVT and its associated complications and consequences in the relevant population, and should include an assessment of patient adherence, both in hospital and after patients have been discharged home. Such a trial would therefore, be very costly to run. In addition, while thigh length stockings appear to have superior efficacy, practical issues may prevent their wide use in clinical practice; patients report that both thigh length and knee length stockings are difficult to use, but fewer patients report discomfort with knee length stockings and patients are more likely to wear knee length stockings correctly.^3–5^ A more pragmatic approach may be to give thigh length stockings only to patients who can use them properly and consistently, while knee length stockings are more appropriate for others.

### Limitations

There was substantial variation across the included trials in terms of the patient characteristics (suggesting that the participants had a different baseline risk for DVT) and interventions used (in terms of both stocking use and concomitant pharmacological prophylaxis). The timing of outcome assessments was generally short, where reported; therefore some DVTs may have been missed. The included trials assessed all DVTs, not just symptomatic DVTs; where reported the majority of DVTs were asymptomatic, the clinical consequences of which are unknown.

Many of the included trials dated back to the 1970s and 1980s, therefore, they may not reflect current practice: surgical practice has changed over time with less invasive surgical procedures, shorter duration of hospitalisation and earlier mobilisation after surgery.

Generally the trial methods were poorly reported, making risk of bias assessment difficult. Only three out of 23 included RCTs were considered to have a low risk of bias; the reporting was inadequate to judge the risk of bias for most trials. This systematic review included all relevant trials, regardless of trial quality; therefore, the uncertain quality of many of the included trials reduces the reliability of the results of this review.

### Conclusions

The evidence base for assessing the relative treatment effectiveness of thigh length and knee length antiembolism stockings for the prevention of DVT in surgical patients is weak; most studies are old and may not reflect current practice.

However, direct and indirect meta-analysis suggests that thigh length stockings may be more effective than knee length stockings, although the results were not statistically significant. Overall, thigh length stockings with pharmacological prophylaxis appears to be the most effective method of preventing DVT in surgical patients, although the marginal benefit of thigh-length stockings plus heparin over heparin alone is less than the marginal benefit of heparin over no treatment as heparin already reduces the risk of DVT substantially.

### Recommendations

Thigh length antiembolism stockings may be more effective than knee length stockings at DVT prevention in surgical patients; however, much of the available research evidence is old and of uncertain quality. A definitive trial in high risk surgical patients to compare thigh length versus knee length antiembolism stockings, in addition to standard pharmacological prophylaxis, would need to be very large to enable assessment of clinically relevant DVT and its associated complications and consequences. Therefore, such a trial would be very costly to run and it is not clear that it would be worthwhile. A more pragmatic approach may be to give thigh length stockings only to patients who can use them properly and consistently, while knee length stockings are more appropriate for patients who are less physically adept or likely to be less compliant.
